# Scientific evolution and translational horizons of plant core germplasm: a global bibliometric synthesis and strategic insights

**DOI:** 10.3389/fpls.2026.1771164

**Published:** 2026-03-19

**Authors:** Wenjun Wang, Hang Ma, Yaodong Qi, Jingxue Ye, Min Lu, Xueping Wei

**Affiliations:** 1 Faculty of Agronomy, Jilin Agricultural University, Changchun, China; 2 State Key Laboratory for Quality Ensurance and Sustainable Use of Dao-di Herbs, Institute of Medicinal Plant Development, Chinese Academy of Medical Sciences & Peking Union Medical College, Beijing, China

**Keywords:** bibliometrics, burst term analysis, core collection, genetic diversity, genome-wide association studies, germplasm, molecular marker

## Abstract

**Introduction:**

The increasing volume of global plant germplasm resources has led to the complexity of plant germplasm resource management. Plant core germplasm collections demonstrate significant potential in improving the efficiency of germplasm resource management, promoting crop improvement, and maintaining biodiversity.

**Methods:**

This study conducted a comprehensive bibliometric analysis of 2,303 Chinese and English publications (2004–2024) sourced from the Web of Science (WoS) and China National Knowledge Infrastructure (CNKI), offering an integrated bilingual perspective on global research patterns and evolution.

**Results and Discussion:**

Results show sustained growth in the field, with China and the United States as the leading contributors and collaborative hubs. The research trajectory progressed through three distinct phases—foundational, consolidation, and predictive—driven by advances from phenotypic evaluation to high-throughput genomics and genome-wide association studies (GWAS). Keyword evolution reveals a clear paradigm shift from descriptive, phenotype-based management toward allele-driven, predictive breeding platforms. Persistent challenges include data fragmentation, limited sharing, and a strong taxonomic bias toward major cereal crops. Looking forward, we propose the integration of artificial intelligence to establish biodigital resource centers and the development of functionally designed core collections tailored to specific plant groups. These strategies will enhance precision breeding and support sustainable agriculture and global food security.

## 1 Introduction

Plant germplasm resources represent the indispensable genetic foundation for agricultural innovation and global food security. The investigation, collection, and evaluation of these resources are prioritized by nations worldwide as the primary determinant of breeding success. According to the *Third Report on the State of the World’s Plant Genetic Resources for Food and Agriculture* (FAO, 2025), there has been an 8% increase in the preservation of seeds and plant materials held in global germplasm collections since 2009, with institutional holdings now totaling approximately 7.4 million accessions (Allender, 2011). For instance, The U.S. National Plant Germplasm System (NPGS) maintains over 600,000 accessions across 454 families (NPGS, 2025). While China’s *Third National Census of Crop Germplasm Resources* has successfully identified and collected 139,000 sets of elite indigenous materials (MARA, 2024).

While this expansion signifies a triumph of biodiversity conservation, it has introduced a “crisis of abundance” that complicates resource management (Chen et al., 2016). The sheer scale of these repositories—often exceeding 50,000 accessions for staple crops like rice—hinders effective characterization and leads to underutilization by breeders who struggle to navigate incomplete passport data and insufficient phenotypic descriptions (Egan et al., 2019a, b). Furthermore, large-scale maintenance is increasingly compromised by resource limitations and human error (Chen et al., 2016). Genetic contamination and accidental loss of rare alleles are particular risks during seed regeneration cycles, where handling practices have been shown to account for nearly 20% of documented taxonomic errors (Girma et al., 2018). Compounding this complexity, an estimated 70% of global accessions are estimated to be redundant, inflating maintenance costs and diluting the efficacy of characterization efforts (Egan et al., 2019b; Sharma et al., 2013).

To bridge this gap between massive *ex situ* preservation and precision utilization, Frankel (1984) introduced the concept of “core germplasm”, defining it as a limited set of accessions representing the maximum genetic diversity of a species with minimum repetitiveness. Brown (1995) introducing the “10% rule,” suggesting that core sets should contain no more than 10% of the total collection to remain manageable. Modern operational frameworks have expanded this concept, prioritizing the creation of “mini-core” collections and objective-driven reference sets to further streamline breeding activities (Razi et al., 2021). Core collections not only maintain population-level genetic diversity but also significantly reduce redundancy and management costs, while streamlining germplasm screening and enhancing breeding efficiency (Oliveira et al., 2010; Belaj et al., 2012). To date, core collections have been successfully established for major cereals (*Oryza sativa* L., *Triticum aestivum* L., *Zea mays* L., *Glycine max* (L.) Merr.) and horticultural species (*Malus pumila* Mill., *Punica granatum* L., *Castanea mollissima* Blume), *Brassica rapa* var. *glabra* Regel, *Solanum tuberosum* L., *Solanum lycopersicum* L., and *Solanum melongena* L.) (Ping, 2008; Gopal et al., 2013; Corrado et al., 2014; Liang et al., 2015; Li et al., 2019; Nie et al., 2021; Sandhu et al., 2022; Wang et al., 2023). These studies provide a robust foundation for the selection of elite varieties.

The construction of core collections has evolved from an early reliance on morphological descriptors—which are often limited by environmental plasticity and seasonal variation —to the integration of high-density molecular markers. Landmark studies have utilized Simple Sequence Repeat (SSR), Random Amplified Polymorphic DNA (RAPD), and Amplified Fragment Length Polymorphism (AFLP) markers to develop stable core sets for species such as yam (*Dioscorea polystachya Turcz.*), pepper (*Capsicum annuum* L.), walnut (*Juglans regia* L.), and olive (*Canarium subulatum*) (Girma et al., 2018; Zewdie et al., 2004; Skroch et al., 1998; Wang et al., 2013; Mouhaddab et al., 2016; Dervishi et al., 2021). Today, the scientific frontier is defined by single-nucleotide polymorphism (SNP) genotyping and genome-wide association studies (GWAS) (Kumar et al., 2015; Brindisi et al., 2023). These technologies have enabled high-resolution translational outcomes, such as the ExHIBiT barley collection for climate resilience (Bernad et al., 2024) and the development of high-yielding rice varieties like Huanghuazhan through purpose-driven core refinement (Zhou et al., 2019).To address management constraints, “mini-core” collections have been developed to target specific breeding goals, such as disease resistance (Mahuku et al., 2003; Pande et al., 2006). Successful mini-cores now exist for chickpea (*Cicer arietinum* L.), sorghum(*Sorghum bicolor* L.), and wheat (*Triticum aestivum* L.) (Upadhyaya et al., 2012; Jiang et al., 2013; Etminan et al., 2017). Despite these advances, the core germplasm research landscape remains fragmented. Taxonomic bias persists, with perennial crops, medicinal plants, and forest trees remaining severely underrepresented compared to major cereals.

The exponential increase in publications on core germplasm has rendered bibliometrics a key instrument for delineating the current landscape and emergent trends of the field. Bibliometrics applies statistical and mathematical techniques to quantitatively dissect large corpora of literature, thereby facilitating an objective assessment of research status and the forecasting of future directions. Quantitative outputs identify prolific authors, map institutional collaborations, and trace technological trajectories, thereby furnishing evidence-based guidance for the optimization of germplasm management strategies (Omer et al., 2025). Previous bibliometric assessments have typically relied on single databases. This study, however, employs a bilingual synthesis of 2,303 publications (2004–2024) from both the Web of Science (WoS) Core Collection and the China National Knowledge Infrastructure (CNKI). While the WoS Core Collection provides a robust framework for international citation networks (Chen et al., 2022). As China is one of the world’s eight centers of crop origin and a leader in current germplasm research, the integration of CNKI encompasses over 95% of Chinese academic journals, capturing indispensable data on indigenous crop history and traditional medicinal plant characterization that are often underrepresented in international databases. We hypothesize that the global research paradigm is shifting from phenotype-based descriptive categorization to high-throughput genomic-led predictive breeding, which necessitates a reimagining of germplasm management. To evaluate this, this study utilizes CiteSpace and VOSviewer to explore the status, hotspots, and frontiers of core germplasm research to guide future development and policy.

## 2 Data collection and methods

### 2.1 Study design and PRISMA framework

**Figure 1:**
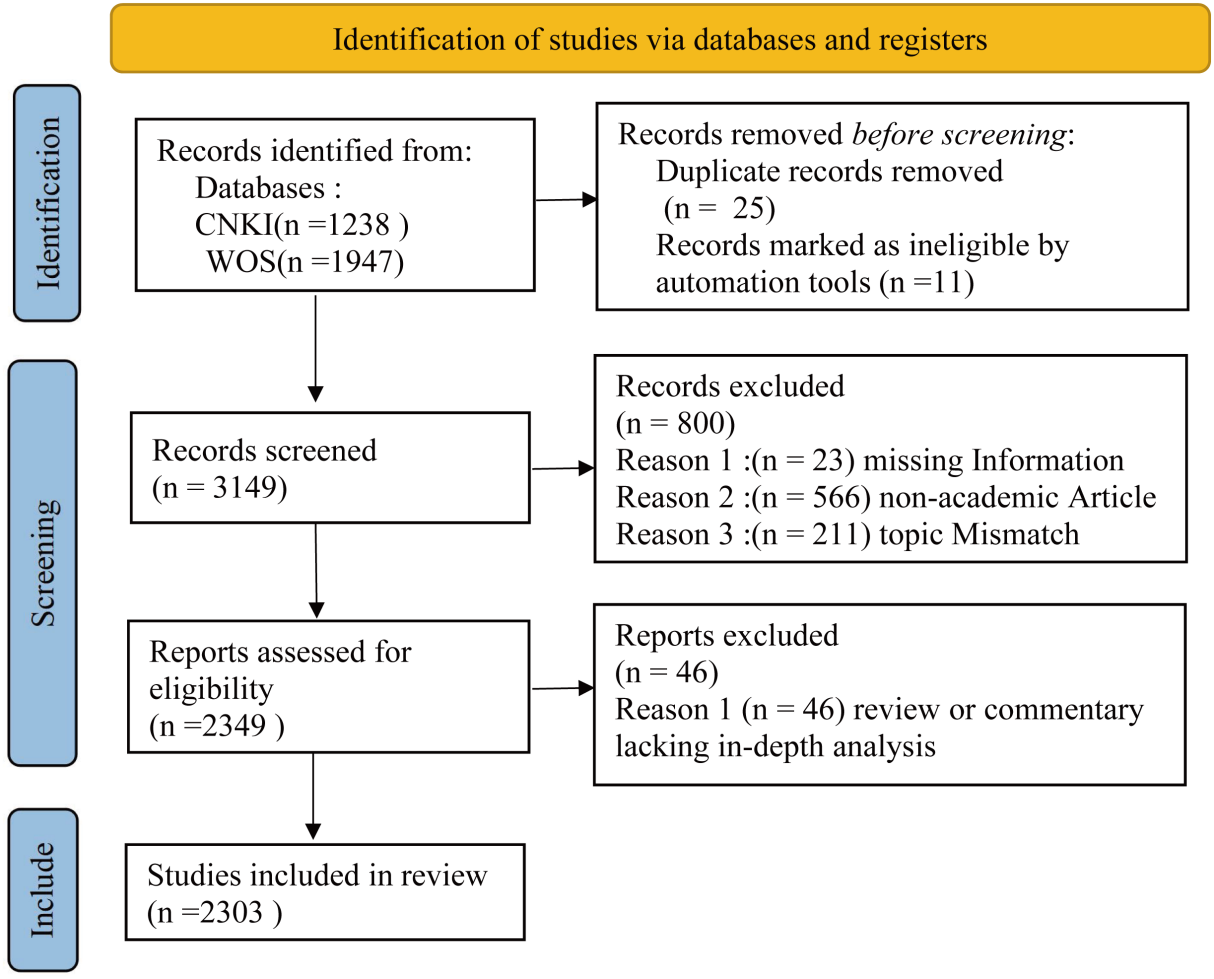
The flowchart shows the methods used for bibliometric analysis in this study.

To ensure transparency and reproducibility, this systematic review followed the PRISMA 2020 (Preferred Reporting Items for Systematic reviews and Meta-Analyses) guidelines (**Figure 1**). The study identifies, screens, and synthesizes global literature on core germplasm published over a 20-year trajectory (2004–2024).

### 2.2 Data sources and retrieval strategy

The selection of WoS Core Collection and CNKI as primary data sources is scientifically justified to overcome the limitations of single-source retrieval. The search was conducted on December 31, 2024, using the following strategies: Chinese Corpus (CNKI): Subject = “核心种质” (Core Germplasm). Document types were limited to academic journals and dissertations. English Corpus (WoS): Topic Search (TS) = (“core collection” OR “core germplasm”) AND TS = (“plant*” OR “crop*”). Document types were limited to “Article” and “Review”. Refinement: Research domains were restricted to Plant Sciences, Agronomy, Genetics & Heredity, Horticulture, Forestry, and Biodiversity Conservation.

### 2.3 Data processing and inclusion criteria

Retrieved records were imported into NoteExpress for deduplication and quality screening. The following inclusion/exclusion criteria were applied: (1) Inclusion: peer-reviewed empirical studies or reviews explicitly addressing the construction, evaluation, or translational application of core and mini-core collections; (2) Exclusion: non-academic items (news reports, conference abstracts), incomplete metadata (missing authors/years), and publications that only peripherally mentioned “core collection” without substantive analysis. The final validated corpus consisted of 2,303 unique records (1,318 from WoS and 985 from CNKI).

### 2.4 Scientometric analysis and visualization tools

The dataset was analyzed using CiteSpace (v6.2.R4) and VOSviewer (v1.6.19)to map the intellectual structure and research frontiers (Chen, 2006; van Eck and Waltman, 2010). The analytical protocol comprised: (1) Publication trend analysis: annual counts were analyzed to delineate the developmental trajectory; (2) Co-occurrence Analysis: Collaborative networks among countries, institutions, and authors were visualized using VOSviewer with “Full Counting” and “Association Strength” normalization; (3) Keyword Dynamics: CiteSpace was utilized for keyword clustering and burst detection to identify hotspots; (4) CiteSpace Parameters: Time slicing = 1 year; Node types = Keyword/Author/Institution; Pruning = Pathfinder/Pruning merged network; network scale controlled by a g-index, K = 5; Threshold = Top 50 most cited or occurring items per slice.

### 2.5 Mathematical modeling of research growth

We applied Mahapatra’s (1985) mathematical model to quantify the field’s “take-off” characteristics (Mahapatra, 1985, 1994). The emergence of research topics is delineated using the Relative Growth Rate (RGR) and Doubling Time (DT). The mean RGR over an interval is calculated as: $\;R(a)=\frac{ln(W2)-ln(W1)}{T2-T1}$, where W1 and W2 are the cumulative number of publications at the beginning and end of the period, and T2−T1 is the time interval. Doubling Time (*D_t_*): *D_t_*= $\frac{0.693}{R(a)}$, this provides an indicator of the years required for a topic’s publication volume to double.

## 3 Results

### 3.1 Publication trend analysis

**Figure 2:**
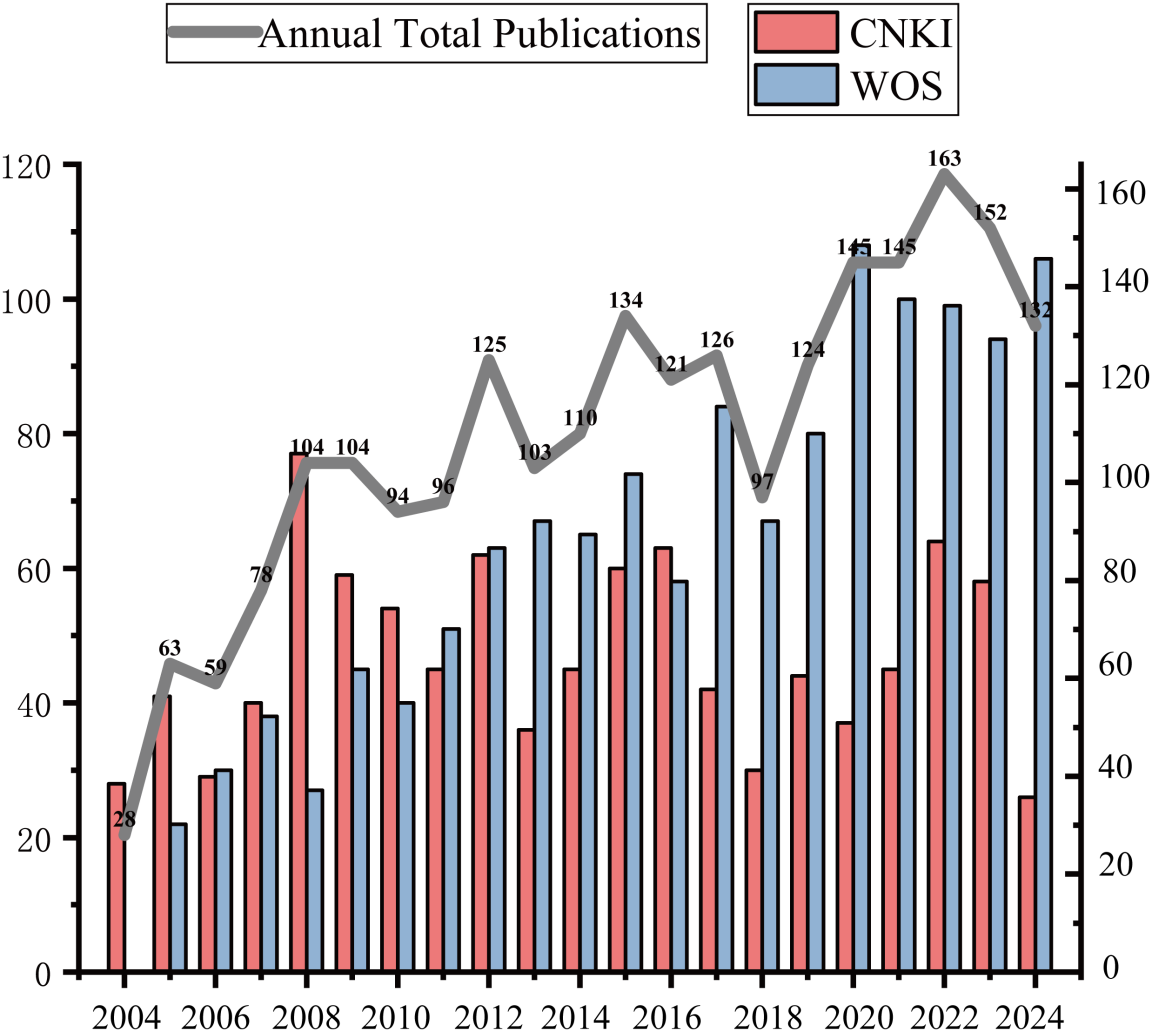
The annual number of publications on core germplasm in WoS and CNKI from 2004–2024.

The annual publication volume from 2004 to 2024 (**Figure 2**) reveals a consistent upward trajectory, signaling the increasing prioritization of core germplasm in global biodiversity and breeding agendas. To move beyond descriptive summation, Mahapatra’s mathematical model showed that the Mean Relative Growth Rate (RGR) for core germplasm literature steady at approximately 0.35 during the initial decade (2004–2014), which was significantly higher than that for 2014–2024 (0.09). the DT for 2004–2014 (2 years) was substantially shorter than that for 2014–2024 (8 years), suggesting that the field gained momentum more rapidly in its earlier phase and has since entered a more stable developmental stage. Notably, in 2008, the RGR reached a minor peak at 33.33%.

### 3.2 Synthesis of landmark studies and research paradigms

The analysis of high-impact literature in both CNKI and WoS (**Supplementary Tables 1**, **2**) identifies a mature research pipeline: Resource Collection →Genetic Diversity Analysis →Core/Mini-Core Construction→Breeding Utilization.

**Figure 3:**
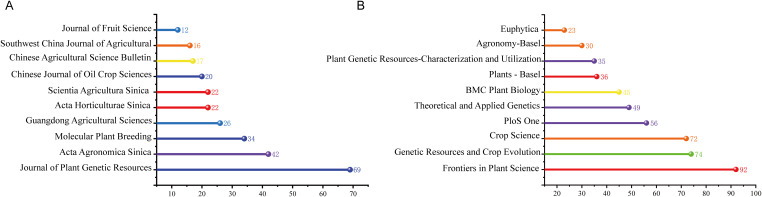
Distribution of publications in core germplasm by journal **(A)** Journal publication data from CNKI **(B)** Journal publication data from WoS.

CNKI Landmark Insights: Standardization and Indigenous Protection. The most-cited Chinese study, Hu et al. (2012) on rice phenotypic diversity, established a standard evaluation framework for major crops in China. These top-cited works collectively emphasize: Phenotypic Robustness: Utilizing multi-trait indicators to ensure core representativeness. Policy Alignment: The 2008 peak in Chinese publications (n=79) aligns with the issuance of Central Document No. 1, which prioritized germplasm innovation. As illustrated in **Figure 3A**, the *Journal of Plant Genetic Resources* published the most articles in the CNKI corpus (n = 69), followed by *Acta Agronomica Sinica* (n = 42). Both journals are recognized as core publications in Chinese agricultural sciences, underscoring the visibility and credibility of core germplasm studies within China’s academic community. The strong emphasis on crop science in these journals further indicates the significant research value and application potential of core germplasm in enhancing germplasm management and cultivar development.

WoS Landmark Insights: The Genomic Revolution. High-impact international literature reflects a shift toward large-scale genomic characterization. The 3,000 Rice Genomes Project was cited over 300 times, this study demonstrated how a core set serves as the foundation for pangenomic analysis and high-resolution SNP discovery (Rellosa et al., 2014). Ma et al. (2018) resequenced a core collection of upland cotton to identify genomic variation influencing fiber quality, illustrating the shift from descriptive statistics to predictive breeding. The top 10 WoS Core Collection journals dedicated to core germplasm research collectively published 512 articles, accounting for 39% of the English corpus (**Figure 3B**). All these journals are ranked in the first quartile (Q1) of the Journal Citation Reports (JCR). *Frontiers in Plant Science* (Switzerland) leads with 92 papers, followed by *Genetic Resources and Crop Evolution* (Germany) with 74. The mean number of citations per article ranges from 5.75 to 49.45, indicating the considerable influence of these journals. Their thematic focus on plant science, genetic resources, and crop evolution reaffirms the central importance of core germplasm in studies of plant genetic diversity.

### 3.3 Author publication volume analysis

**Figure 4:**
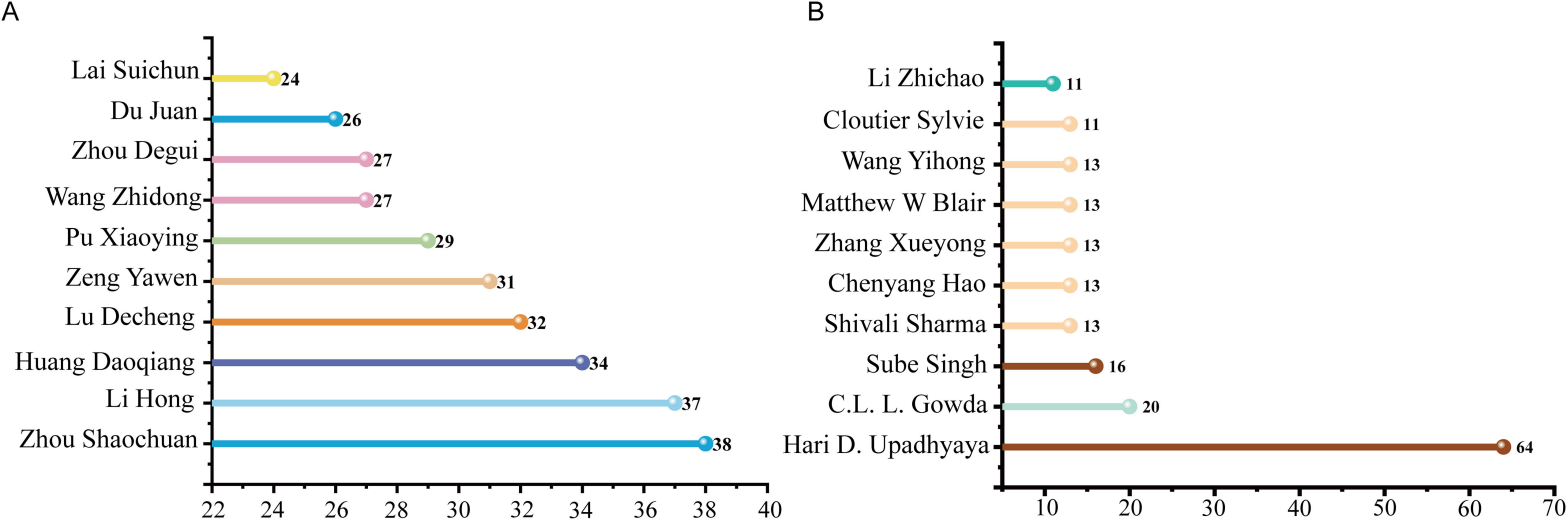
Productivity of leading scholars in core germplasm studies **(A)** The most prolific authors in the CNKI database **(B)** The most prolific authors in the WoS.

Author-level productivity metrics help identify leading researchers and collaborative networks in the field of core germplasm. As shown in **Figure 4B**, the ten most prolific English-language authors are led by Hari D. Upadhyaya (University of Georgia/International Crops Research Institute for the Semi-Arid Tropics (ICRISAT)), who published 64 articles on core germplasm between 2004 and 2024. These works have received a total of 1980 citations, with an average of 31 citations per article. His studies focuses on *Cicer arietinum* L. and *Arachis hypogaea* L., with an emphasis on resistance breeding, genetic diversity analysis, targeted germplasm screening and the identification of beneficial alleles (Upadhyaya and Ortiz, 2001; Kashiwagi et al., 2005; Upadhyaya et al., 2007a, b; Kashiwagi et al., 2013; Upadhyaya et al., 2016; Varshney et al., 2021). The second most prolific author is C.L.L. Gowda from ICRISAT, with a total of 20 publications. The average citation frequency per article is 20. His research focuses on crop improvement, with chickpea being the primary subject. His work primarily involves the screening of superior germplasm materials for crop improvement, analysis of genetic diversity, and identification of candidate genes related to these traits (Das et al., 2015). **Figure 4A** summarizes the Chinese corpus: 2,841 authors published in this field from 2004 to 2024, among whom 85 published five or more papers. The most productive researchers—Zhou Shaochuan (38 papers), Li Hong (37) and Huang Daoqiang (34)—concentrated on the development, comprehensive evaluation and efficient exploitation of rice core collection (Zhou et al., 2021; Huang et al., 2022).The collaboration network (**Supplementary Figure 1**) shows that internal collaboration within domestic and international research teams is relatively close, but the connections between the groups are relatively scattered.

### 3.4 Country-level publication output

**Figure 5:**
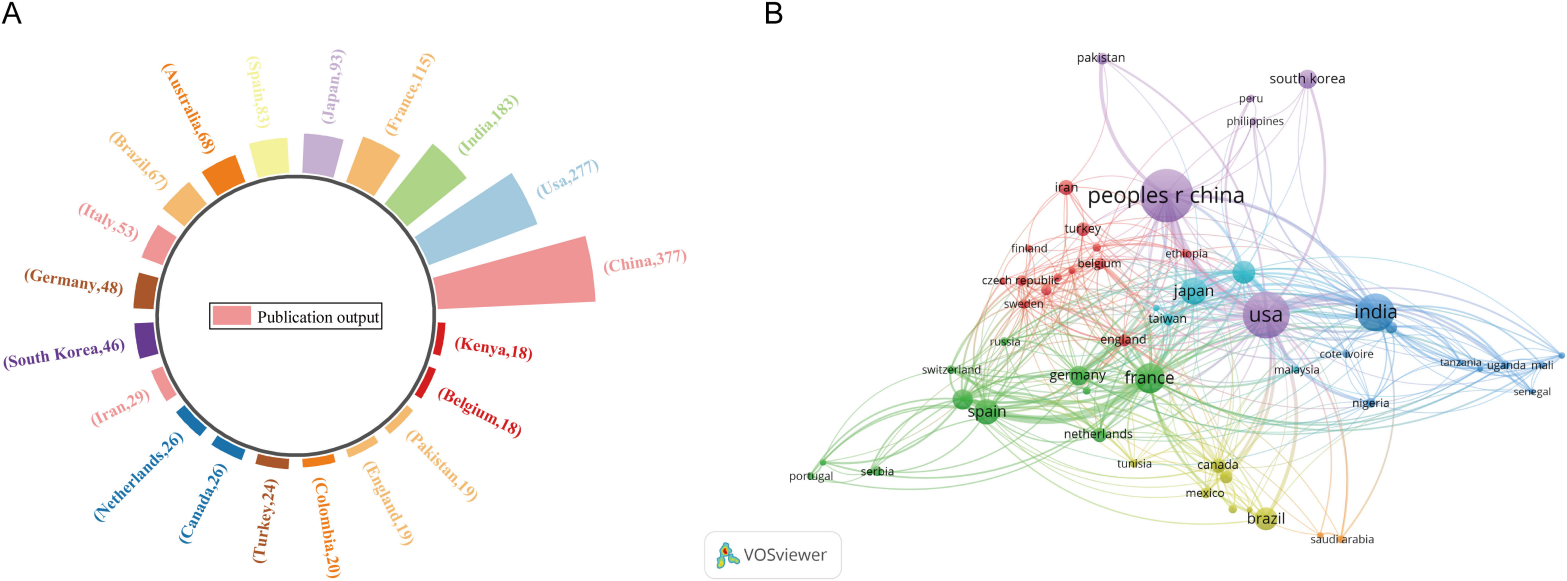
International research output in the field of core germplasm **(A)** Proportion of articles from the top 20 most prolific countries **(B)** Country co-authorship network for core germplasm publications.

Analysis of the WoS data indicates that the top 20 most productive countries collectively contributed 1,609 articles—a number exceeding the total count of retrieved records, reflecting a high degree of international co-authorship in the field. As illustrated in **Figure 5A**, China was the leading contributor with 377 publications, followed by the United States (n = 277) and India (n = 183). underscoring China’s prominent role in core germplasm research. The collaboration network map (**Figure 5B**) further demonstrates that China maintains strong cooperative links with the United States, India, France, and Japan. The extensive interconnectivity observed among most countries reveals a multifaceted and collaborative global research architecture.

### 3.5 Institutional output analysis

**Figure 6:**
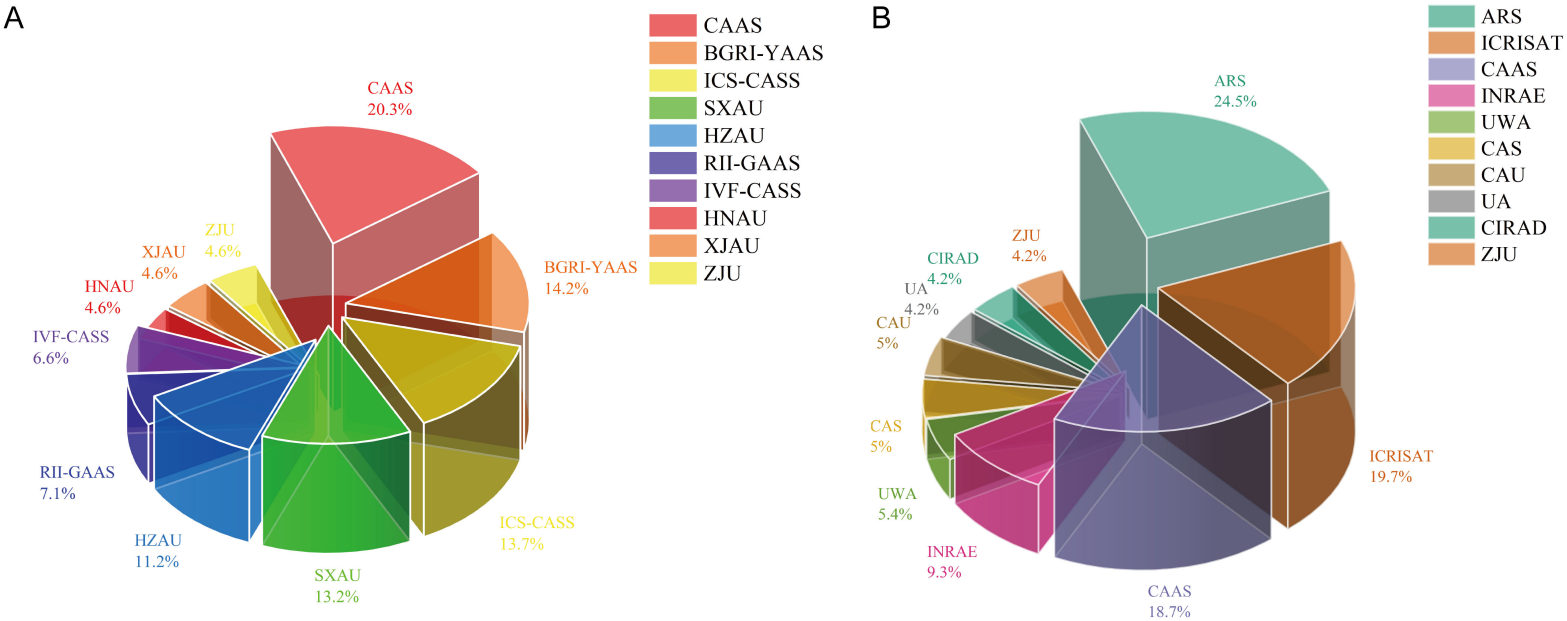
Scientific output of major institutions in the core germplasm research field **(A)** Top 10 institutions by publication count in the CNKI **(B)** Top 10 institutions by publication count in the WoS.

The distribution of publications among institutions highlights distinct patterns between Chinese and global contributors. Within the CNKI corpus, the ten most prolific institutions are primarily universities and research academies, led by the Chinese Academy of Agricultural Sciences with 40 articles (**Figure 6A**). In contrast, 47 institutions in the WoS Core Collection have published more than ten articles each, indicating a wider and more diverse global institutional engagement (**Figure 6B**). Notably, four of the top ten most productive institutions worldwide are from China, collectively contributing 165 articles. The collaboration network (**Supplementary Figure 2**) shows that a core has currently been formed, consisting of a few key units including the Agricultural Bureau of the United States Department of Agriculture, the Chinese Academy of Agricultural Sciences (CAAS), and the International Crops Research Institute for the Semi-Arid Tropics (ICRISAT). These core units exhibit high centrality. This also demonstrates that these institutions’ research in the field of core germplasm holds guiding significance, and they maintain close collaborative relationships with other institutions.

### 3.6 Keyword analysis

#### 3.6.1 Keyword co-occurrence analysis

**Figure 7:**
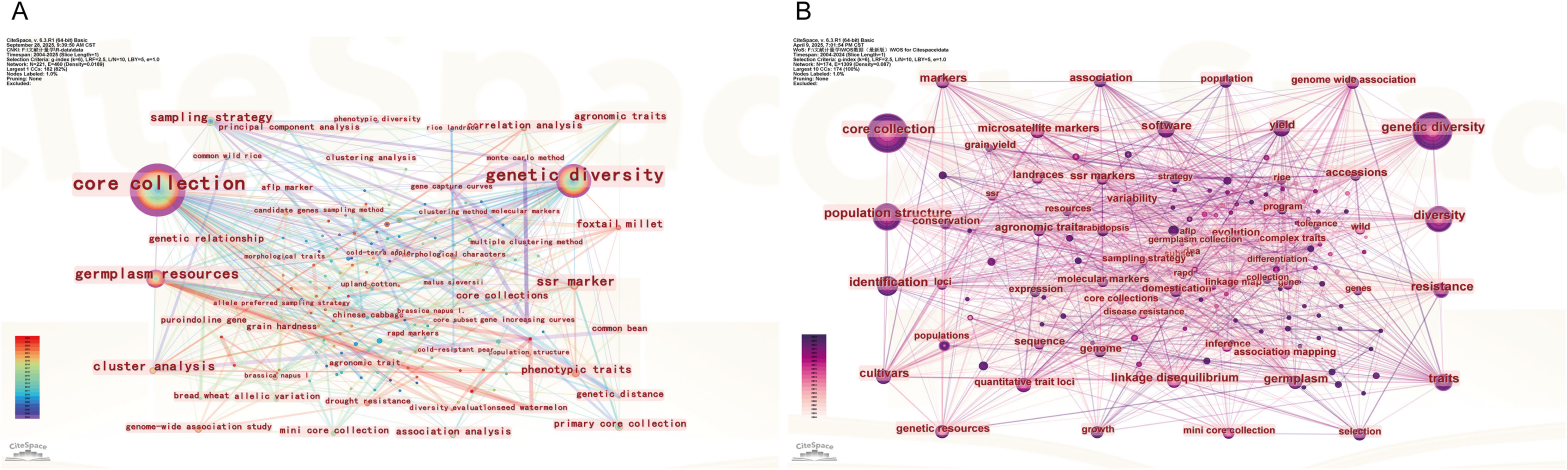
Keyword co-occurrence analysis on core germplasm **(A)** Keywords of Chinese literature **(B)** Keywords from English-language literature.

Keyword co-occurrence mapping was employed to identify high-frequency and high-centrality terms, delineating research hotspots within the core germplasm field (**Table 1**). This approach offers a comprehensive perspective on emerging trends and frontiers thereby helping to guide future research priorities (Ge et al., 2024). In the WoS corpus the most frequent terms were “genetic diversity” (389), “core collection” (328) and “population structure” (232), indicating that international research emphasizes germplasm diversity, trait discovery and the application of molecular-marker (**Figure 7B**). In the Chinese corpus (CNKI) (**Figure 7A**), the predominant keywords included “core collection” (533), “genetic diversity” (263), “germplasm resources” (117), “cluster analysis” (40), “phenotypic traits” (30) and “agronomic traits” (24). The research focus in Chinese literature centers on methodologies for constructing core collections—such as cluster analysis and sampling strategies—as well as diversity assessments of field-crop germplasm and multi-trait phenotypic evaluations.

**Table 1:** Top 20 keywords on core germplasm in WoS and CNKI.

CNKI			WoS		
No.	Key words	Frequency	No.	Key words	Frequency
1	Core collection	533	1	Genetic diversity	389
2	Genetic diversity	263	2	Core collection	328
3	Germplasm resources	117	3	Population structure	232
4	Cluster analysis	40	4	Diversity	196
5	Ssr marker	39	5	Identification	167
6	Phenotypic traits	30	6	Germplasm	121
7	Agronomic traits	24	7	Cultivars	117
8	Primary core collection	24	8	Resistance	111
9	Sampling strategy	22	9	Traits	111
10	Genome-wide association study	12	10	Markers	109
11	Association analysis	11	11	Software	102
12	Foxtail millet	10	12	Linkage disequilibrium	91
13	Mini core collection	9	13	Genetic resources	78
14	Sampling methods	8	14	Yield	76
15	Cluster method	7	15	Genome-wide association	74
16	Correlation analysis	7	16	Quantitative trait loci	68
17	Population structure	7	17	Ssr markers	61
18	Genetic distance	6	18	Association	57
19	Common bean	6	19	Genome	52
20	Allelic variation	5	20	Microsatellite markers	52

**Figure 8:**
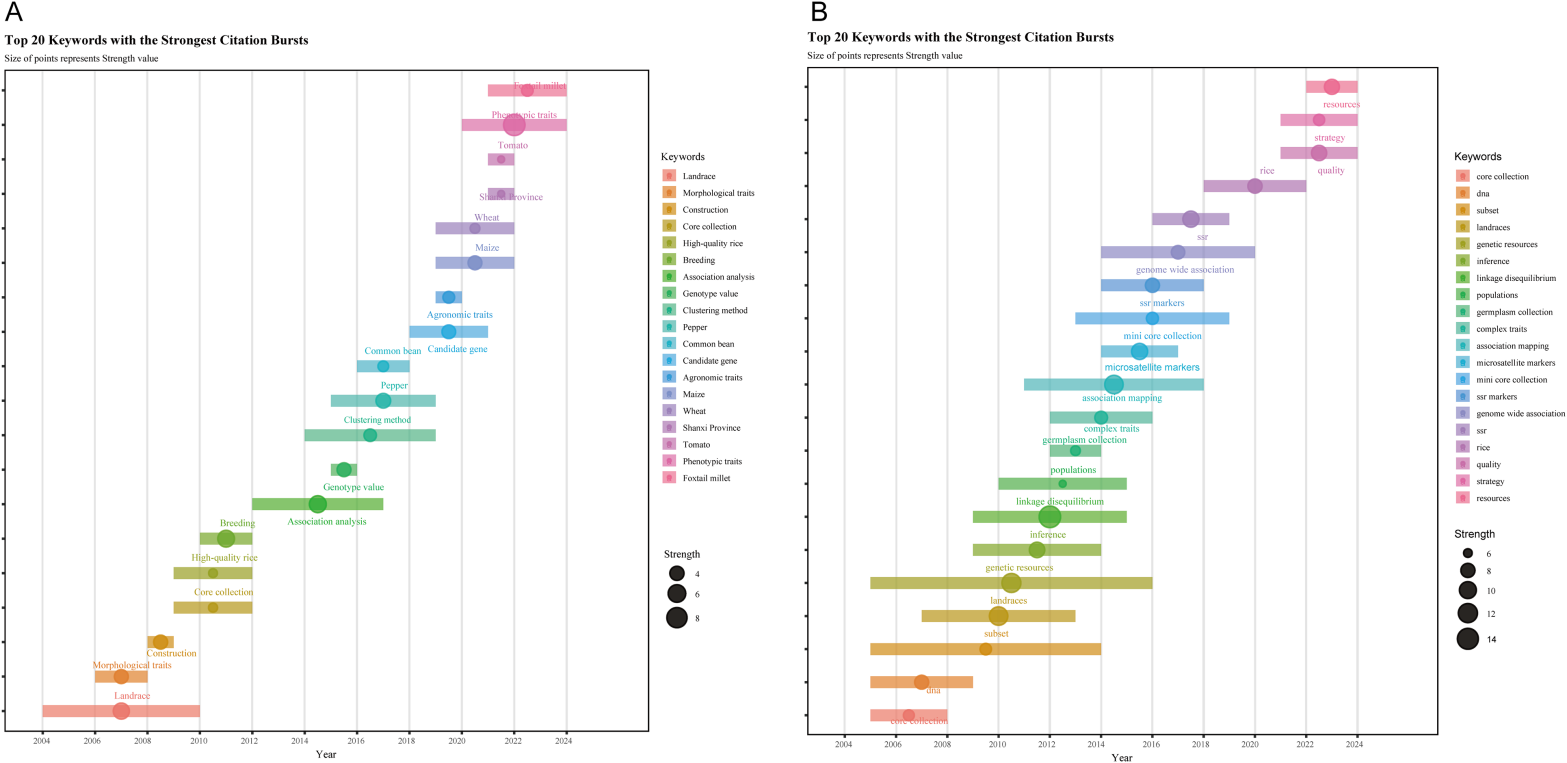
Keyword Prominence analysison core germplasm **(A)** CNKI **(B)** Web of Science (WoS).

**Figure 9:**
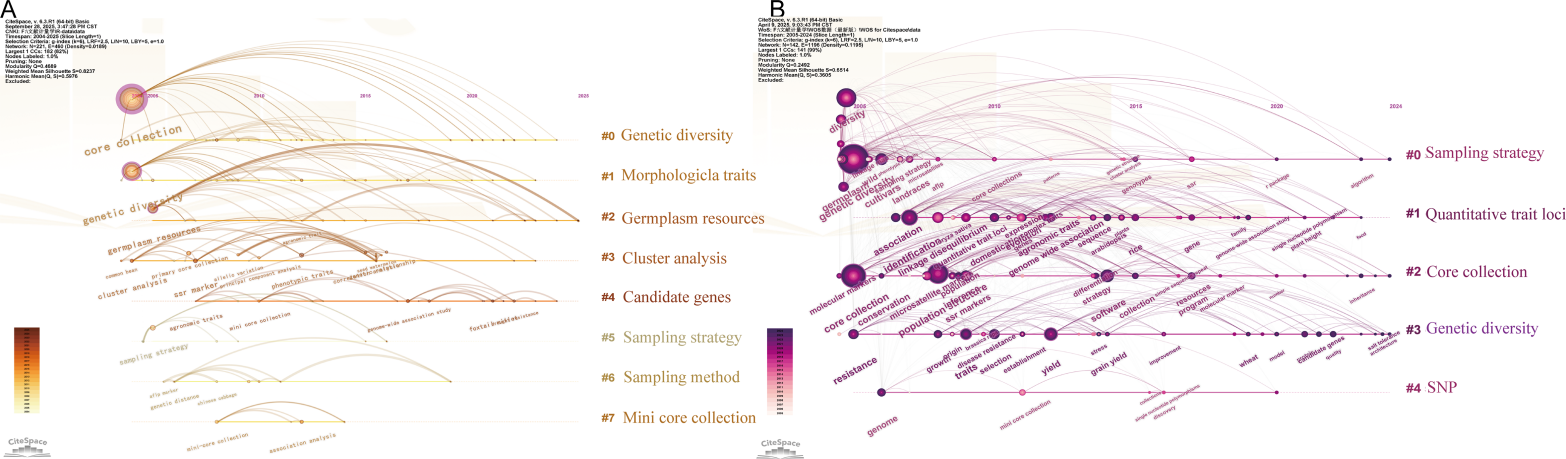
Keyword Timeline Chart on core germplasm **(A)** CNKI **(B)** WoS.

Keyword co-occurrence and burst analysis (**Figures 7**–**9**) delineate the field’s transition from morphological to functional genomics. Foundational Phase (2004–2011): This period was characterized by dominant bioconcepts centered on genetic distance, cluster analysis, and SSR markers. The primary technological drivers were morphological descriptors and PCR-based molecular markers. The overarching strategic goal focused on resource rationalization of germplasm collections. Consolidation Phase (2012–2017): Research emphasis shifted towards bioconcepts such as population structure and linkage disequilibrium. Technologically, the field was propelled by the adoption of SNP arrays and heuristic algorithms. The key strategic objective evolved to achieving objective representativeness in core collection development. Predictive Phase (2018–2024): The current era is defined by bioconcepts including GWAS, candidate gene identification, and climate resilience. It is driven by advanced technologies such as whole-genome sequencing (WGS) and Artificial Intelligence/Deep Learning. The strategic goal has decisively moved towards translational cultivar release, bridging genomic discovery with practical breeding outcomes. The time-zone map (**Figure 9**) shows that while “phenotypic traits” maintains sustained intensity, keywords such as “genotype value” and “KASP (Kompetitive Allele-Specific PCR) markers” have emerged as the current research frontiers.

We conducted a statistical analysis of the Relative Growth Rate (RGR) and Doubling Time (DT) for burst terms. The results indicate that while genetic diversity and germplasm resources serve as the primary research themes in the core germplasm field with high centrality, SNP and GWAS, as emerging burst terms, have exhibited a significantly accelerated RGR since 2014. Specifically, the Doubling Time (DT) for SNP has decreased sharply from 26 years (2004–2014) to 7 years (2014–2024), a trend closely associated with the widespread adoption of high-throughput sequencing technologies. Notably, the RGR for phenotypic traits—fundamental data in germplasm research—has declined from an initial 0.0736 to 0.019, aligning with the hotspot analysis in core germplasm research.

#### 3.6.2 Keyword clustering analysis

Keyword clustering of both Chinese and English corpora was conducted to elucidate dominant research trajectories and thematic focuses in core germplasm science. The Chinese-language keyword clusters, detailed in **Table 2**, can be broadly categorized into two overarching streams:

**Table 2:** Keyword clustering analysis of core germplasm studies in CNKI.

Cluster name	Size	Year	Top keywords
#0 Core collection	57	2012	breeding, agronomic performance, agronomic trait
#1 Genetic diversity	34	2011	population structure, aflp, ssr, allele preferred sampling strategy
#2 Germplasm resources	24	2014	rapd markers, core primers, diversity evaluation
#3 Cluster analysis	19	2011	phenotypic traits, common bean, upland cotton
#4 Sampling strategy	16	2007	multiple clustering method, monte carlo method, gene capture curves
#5 Mini core collection	15	2012	association analysis, allelic variation, geographic core collection
#6 Agronomic traits	14	2008	ecological difference, booting stage, Yunnan rice landrace
#7 Candidate genes	13	2019	drought resistance, salt tolerance, genome-wide association study

Stream I—Methodology & Resource Basis: Cluster #2 (germplasm resources) represents the fundamental genetic material and primary subject of study. Cluster #4 (sampling strategy) addresses the methodological approach for selecting representative accessions from extensive germplasm pools, forming the basis for subsequent analyses. Cluster #1 (genetic diversity) provides the analytical metrics required to validate the representativeness of selected samples; outputs from Cluster #4 are evaluated using methods from Cluster #1. Serving as the thematic hub, Cluster #0 (core collection) integrates inputs from Clusters #4, #1, and #3 to assemble a compact yet highly representative panel, intended as a miniaturized but genetically comprehensive resource for further research. This panel is often further refined into a mini-core collection (Cluster #5). The core germplasm construction pipeline generally involves: (i) estimating genetic distance, (ii) selecting clustering algorithms, (iii) optimizing sampling ratios, (iv) implementing the sampling strategy, and (v) performing a comprehensive evaluation. Multivariate techniques, primarily cluster analysis and principal component analysis, are routinely applied to capture both representative and unique accessions while quantifying their genetic diversity. The choice of sampling strategy and ratio is critical, as it directly affects the allelic representativeness of the final collection; empirically, larger base collections typically require a lower sampling fraction to capture allelic richness, whereas smaller collections need a higher fraction to prevent diversity loss.

Stream II—Molecular Mechanisms: Cluster #7 (candidate genes) utilizes genome-wide association studies (GWAS) conducted on core or mini-core collections to rapidly identify loci governing important agronomic traits such as drought and salt tolerance. This stream represents the current research frontier in the discipline.

A similar clustering analysis of the English-language corpus (**Table 3**) reveals a coherent thematic structure. Accessions are initially processed through Cluster #0 (sampling strategy) to establish a representative Cluster #2 (core collection). The genetic diversity of this core set is then characterized using methods associated with Cluster #3 (genetic diversity). Notably, Cluster #4 (SNP) highlights the impact of high-throughput, next-generation molecular markers. SNP technology has substantially advanced gene-mapping research by enhancing the capacity for genetic diversity assessment, thereby acting as a major technological driver in modern core germplasm construction (Wu et al., 2024). Collectively, the keyword clustering analyses of both corpora document a clear paradigm shift within core germplasm science, from traditional resource description toward modern molecular breeding.

**Table 3:** Keyword clustering analysis of core germplasm studies in WoS.

Cluster name	Size	Year	Top keywords
#0 Sampling strategy	40	2008	genetic differentiation, oryza sativa, mini core collection, genetic resources
#1 Quantitative trait loci	36	2013	association mapping, genome wide association, kasp, linkage disequilibrium
#2 Core collection	29	2013	sequence-specific marker, population structure, ssr markers, microsatellite markers
#3 Genetic diversity	29	2015	agronomic traits, trait-specific germplasm, candidate genes
#4 SNP	7	2013	gene pools, qtl analysis, population structure

#### 3.6.3 Analysis of key research areas in core germplasm studies

The temporal distribution and emergence patterns of keywords help delineate the evolutionary trajectory and research frontiers in core germplasm studies. In the timeline visualization, the X-axis denotes the first appearance year of keywords within a cluster, while the Y-axis indicates their cluster affiliation, thereby illustrating the emergence, development, and temporal dynamics of each thematic cluster. Algorithmic burst detection was applied to identify keywords that experienced a sudden or sustained surge in prominence within specific time windows. Salience and time-zone mapping based on the co-occurrence matrix (**Figure 8A**) identified early-emerging, high-salience terms in the Chinese corpus such as “sampling strategy,” “selection and breeding,” and “high-quality rice.” These terms reflect an initial research emphasis on methodological foundations and conventional breeding, aimed at identifying representative accessions for cultivar development. For instance, Xu et al. (2004) compared different genetic distances, clustering methods, and sampling schemes to construct a cotton core collection, while Li et al. (2005) utilized rice core collection to develop elite, high-quality cultivars. Subsequent burst detection identified “genotype value” as a high-intensity keyword. The time-zone map (**Figure 9A**) illustrates a technology-driven transition from morphological to molecular genetics paradigms. It is noteworthy that “phenotypic traits” maintains a sustained burst intensity in the Chinese literature, reflecting the widespread integration of high-throughput phenomics and digital imaging platforms.

Time-zone and burst analysis of the English-language articles (**Figures 8B**, **9B**) reveals two distinct research phases. Early high-burst terms, including “genetic resources” and “landraces,” characterize an initial phase focused on germplasm conservation. Landraces, developed through centuries of local cultivation, harbor unique allelic diversity that serves as a valuable reservoir for germplasm innovation (Freitag et al., 2012). For example, the evaluation of a Spanish common-bean core collection uncovered substantial agronomic diversity among landraces (Pérez-Vega et al., 2009). The rise of genome-wide association studies (GWAS) as a dominant analytical framework has brought terms like “mini core collection,” “quality,” and “strategy” into focus, as indicated by burst analysis. This trend is supported by the widespread adoption of high-throughput sequencing, which enables large-scale genotype–phenotype association scans (Jia et al., 2021). The application of GWAS to a pepper core collection, for example, has identified resistance loci now used in breeding programs (Brindisi et al., 2023). This evolution underscores a broader paradigm shift toward allelic stewardship and the sustainable utilization of germplasm resources. The trajectory evident in English-language publications progresses from *ex situ* conservation, through molecular characterization (including marker profiling and GWAS) and allele mining, toward the final deployment of core germplasm in breeding.

### 3.7 Statistical analysis of core germplasm applications in plant science

**Figure 10:**
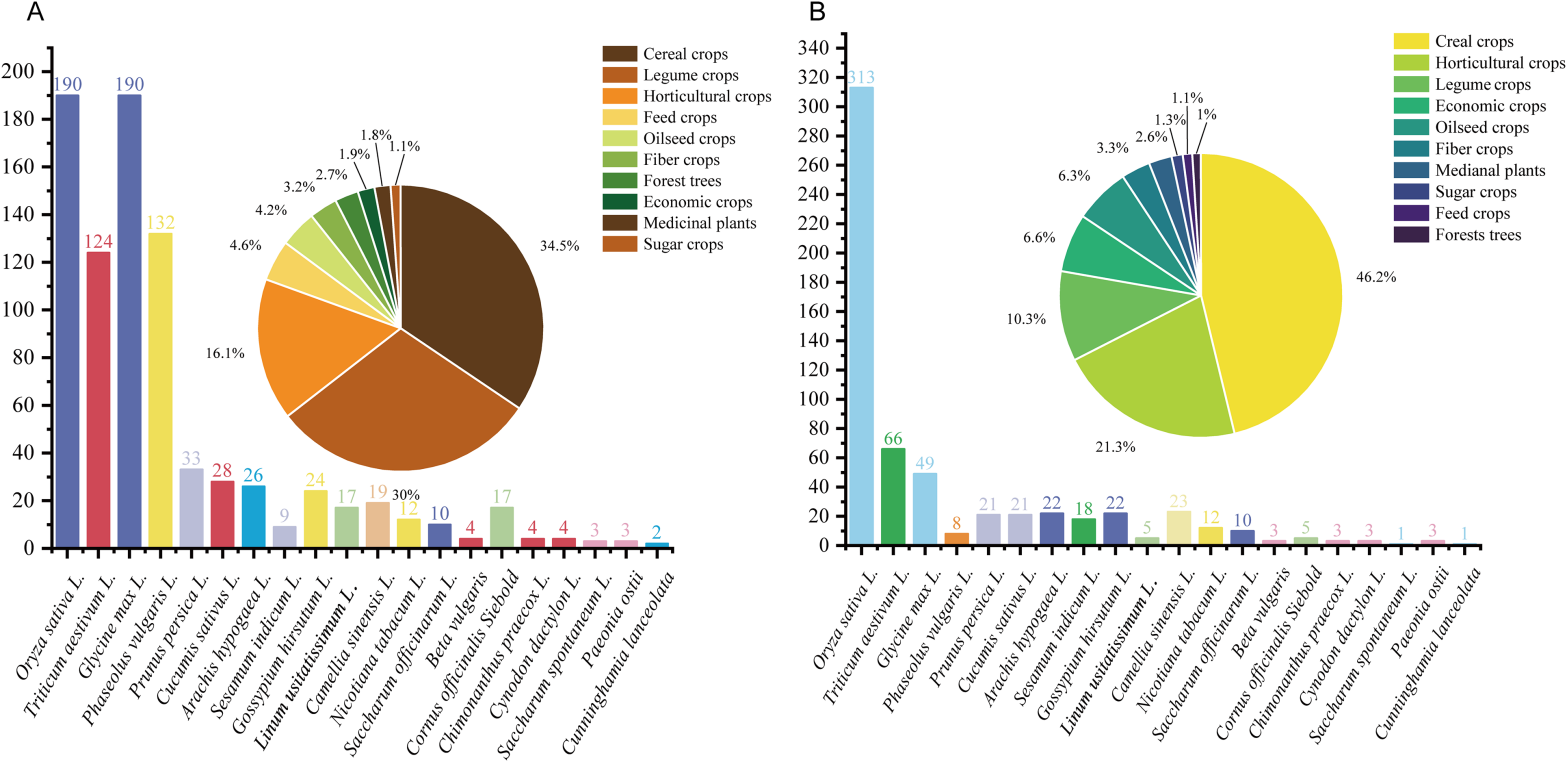
Research frequency of plant with established core collections. (**A)** Species and publication frequency based on the WoS (English) database. **(B)** Species and publication frequency based on the CNKI (Chinese) database. The pie chart in the upper-left corner illustrates the proportion of major crop types represented in the studies. Species are categorized and color-coded by crop group. Bar height corresponds to the number of studies reporting core collection development for each species. Data cover publications from 2004 to 2024.

A statistical assessment of plant groups (**Figure 10**) reveals a significant taxonomic bias. Cereal crops (rice, wheat, maize) dominate the landscape (~46.2% of CNKI corpus), reflecting their strategic importance for food security. Studies on core germplasm are predominantly concentrated on a limited number of major staple crops. Rice (*Oryza sativa* L.) and wheat (*Triticum aestivum* L.) account for the largest share of research output, reflecting their paramount strategic importance for global food security and the prioritized allocation of germplasm resources. In contrast, taxa such as forest trees, medicinal plants, and sugar crops are substantially underrepresented in both literature corpora. These groups, critical for biodiversity conservation, ecosystem services, and new resource development, constitute clear priorities for future research intensification.

Notable differences emerge at the plant-group level between the two literature sets. In English-language publications, legumes—including soybean (*Glycine max* L.) and common bean (*Phaseolus vulgaris* L.)—receive research attention comparable to that of cereals. Conversely, Chinese-language literature includes notably fewer studies on legumes, with horticultural crops (i.e., vegetables and fruits) representing the second-largest research category. This disparity likely reflects regional variations in agricultural demands, dietary patterns, and industry structures.

### 3.8 A complete framework from collection to cultivar

Research has been most systematic and has established a complete, transferable model for major food crops possessing abundant germplasm resources, with rice serving as the foremost exemplar. The work on this crop has established a comprehensive “construction → evaluation → gene mining → cultivar development” pipeline.

In the construction phase, methodological advancements are evident. A Chinese research team constructed a primary core collection of 4,000 accessions from 50,526 locally stored samples by analyzing 26 phenotypic traits and applying a specialized logarithmic/square-root ratio random-sampling strategy based on the Ding Ying taxonomic system. This work also proposed a three-tier refinement framework (‘phenotype → molecular/physiological → final selection’), establishing a standardized procedure for large-scale germplasm management (Li et al., 2003). Complementing this, an international study by Agrama et al. (2009) integrated 26 phenotypic traits and 77 SSR markers from 1,794 USDA rice accessions, utilizing PowerCore’s heuristic algorithm to enhance scientific rigor and representativeness.

Subsequent studies have robustly validated the utility of these core collections for downstream applications. For instance, Xia et al. (2014) evaluated cold tolerance in a Guangxi landrace rice core set to identify resistant accessions, while Li et al. (2011) mapped 30 yield-related QTLs in a U.S. micro-core collection for direct application in marker-assisted selection. Furthermore, high-yielding varieties, including Huanghuazhan, Huangsizhan, and Huanglizhan, have been successfully developed from core germplasm, significantly shortening breeding cycles (Zhou et al., 2019). Collectively, these efforts demonstrate a mature and highly effective paradigm for translating conserved genetic diversity into breeding gain.

## 4 Discussion

Based on an bibliometric analysis of 2303 Chinese and English publications from 2004 to 2024, coupled with the latest global developments in germplasm resources in 2025, it can be observed that research on plant core collections is at a critical historical juncture, transitioning from a “supporting tool for resource management” to a “discovery engine for precision breeding.” This evolution not only reflects the technological dividends brought about by declining sequencing costs but also demonstrates a strategic shift in global priorities in addressing food security, climate change, and biodiversity loss.

### 4.1 Evolutionary stages and regional landscape of global core germplasm development: from conceptual foundation to technology-driven integration

The first two decades following the introduction of the core germplasm concept (circa 1984–2004) were primarily a conceptual and exploratory phase, during which foundational methodologies were established. Our bibliometric analysis, focusing on 2004–2024, delineates subsequent distinct phases driven by technological advances and policy initiatives (**Figure 2**). The research trajectory over the past two decades can be clearly divided into three stages. The first stage (approximately 2004-2011) was a foundational period for methodology, focusing on validating the applicability of the core germplasm concept across different crops and exploring sampling strategies based on phenotypic data. The second stage (2012-2017) was driven by molecular markers, where the application of SSRs, AFLPs, and early SNP chips significantly enhanced the genetic representativeness of core collections. The third stage (2018 to present) has entered the era of genomic selection and digital germplasm, where core collections are being redefined as training populations for association analysis and whole-genome prediction.

Globally, China has emerged as the most prolific contributor (377 publications), followed by the United States and India (**Figure 5A**), reflecting its vast germplasm resources and strategic investment reflecting massive national investment in germplasm resources. In terms of geographical distribution, China ranks first globally in terms of publication output, which is closely related to its vast germplasm holdings and sustained policy investments. China’s Third National Survey of Crop Germplasm Resources collected 139,000 accessions, providing a solid material foundation for core collection construction. However, analysis of international collaboration networks (**Figure 5B**) reveals that despite China’s high output, international organizations under CGIAR (such as ICRISAT, CIP, CIAT) remain central hubs for theoretical innovation and setting global standards.

### 4.2 Informativeness and methodological efficacy: from phenotypes to genomes

A critical question for breeders and curators is whether constructed core collections are genetically informative and which methodological frameworks prove most effective for modern breeding. Our keyword and clustering analyses (**Tables 1**-**3**, **Figures 7**-**9**) delineate a clear, technology-driven evolutionary trajectory in the field, which can be summarized into three main phases: the phenotype-dominant period, the molecular marker period, and the current genomics and association analysis period.

Early research (pre-2010) was dominated by phenotype-centric strategies (Wang et al., 2008). For instance, Girma et al. constructed a yam core collection using 56 phenotypic descriptors (Girma et al., 2018), while Zewdie evaluated pepper resources based on 21 traits (Zewdie et al., 2004). Although phenotypic data are intuitive and possess practical breeding significance, their limitations—such as susceptibility to genotype-by-environment (G×E) interactions—drove the field towards more objective molecular markers (Skroch et al., 1998; Wang et al., 2013; Mouhaddab et al., 2016; Dervishi et al., 2021). Lee et al. (2016) constructed a core collection of pepper germplasm resources based on 48 SNP markers and 32 morphological traits. This approach not only captured a broad range of phenotypic variation but also exhibited higher genetic diversity, thereby providing a key resource for pepper breeding and genetic association analysis. The current methodological frontier is now propelled by “whole-genome resequencing (WGS).” For example, the “3,000 Rice Genomes Project” has elevated the representativeness of core collections from the level of allele frequencies to encompass structural variants (SVs) and presence-absence variations (PAVs) through large-scale sequencing (Rellosa et al., 2014; Wei et al., 2021). Gouy et al. (2025) employed GWAS and genomic selection (GS) to conduct genetic analysis and optimization on a global flax germplasm resource comprising 1,593 accessions from 42 countries, ultimately successfully constructing a representative core collection of 409 accessions, and demonstrated that the strategy combining the Shannon index with allelic coverage (SH+CV) is most effective in balancing genetic diversity, representativeness, and QTL detection capability. This integration of high-dimensional data is transforming core collection construction from a “sampling art” into a “computational science”.

The core algorithms underpinning this evolution are varied (**Table 4**): the M-strategy (Maximization) prioritizes maximizing allelic richness, favoring the discovery of rare variants but potentially at the cost of phenotypic balance, implemented by tools like PowerCore and MSTRAT (Kim et al., 2007; Gouesnard et al., 2001); Multi-Objective Optimization (MOO) simultaneously optimizes diversity and representativeness, balancing genetic distance and phenotypic coverage albeit with higher computational complexity, exemplified by CoreHunter 3 (De Beukelaer et al., 2018; Chen et al., 2025b); the Least distance stepwise sampling (LDSS) strategy employs linkage disequilibrium-based sampling to significantly enhance the sensitivity of QTL mapping, though it depends on high-density SNP maps and often requires custom scripts (Gouy et al., 2025); finally, Prediction-Informed Sampling selects accessions based on genomic estimated breeding values, enabling targeted screening for specific stress resistance traits, yet necessitates high-quality training datasets, with tools like TrainSel facilitating this approach, such as the study of Yuan et al. (2026), they employed a combined approach of genome-wide prediction (GWP) and GWAS, utilizing tools such as TrainSel for core collection optimization, to conduct large-scale prediction, field validation, and genetic dissection of disease resistance traits in 20,458 barley accessions from the German genebank, thereby enabling targeted screening for specific stress resistance traits.

**Table 4:** The status of partial core collection development in plant science.

Type	Name	Data	Method	Ratio	Software	References
Cereal crops	*Oryza sativa* L.	phenotypic traits	Dingying’s taxonomic system+square root + logarithm + random sampling	8%	Programming with FoxPro	(Li et al., 2003)
	*Oryza sativa* L.	phenotypic traits+SSR	PowerCore	12%	PowerCore	(Agrama et al., 2009)
	*Triticum aestivum* L.	SSR	‘M’ strategy	9%	MSTRAT	(Balfourier et al., 2007)
	*Hordeum vulgare* L.	phenotypic traits	UPGMA+priority sampling	10%	NT-SY Spc	(Xu et al., 2011)
Legume crops	*Glycine max* L.	phenotypic traits+SSR	‘S’strategy (hierarchical clustering+square rootstrategy)	2%	NT-SY Spc	(Wang et al., 2006)
	*Pisum sativum* L.	SSR	UPGMA+random sampling	12%	NT-SY Spc	(Zong et al., 2009)
Horticultural crops	*Solanum lycopersicum* L.	Phenotypic traits+SNP	Phenotypic:Mahalanobis distance + 10% + Preferred sampling + Weighted pair-group average method SNP:Core Hunter-20%	28%	Phenotypic:QGA-station SNP: Core Hunter	(Chen et al., 2025b)
Oilseed crops	*Arachis hypogaea* L.	phenotypic traits	Phenotypic:Euclidean distance+the shortest distance method+priority sampling	50%	QGA-station	(Mu et al., 2017)
	*Linum* *usitatissimum* L	phenotypic traits+SNP	mixed strategy: Breeder’s selection of important materials+Shannon index combined with the allelic coverage	26%	CoreCollection corehunter	(Gouy et al., 2025)
Medicinal plants	*Cornus officinalis* Siebold & Zucc.	phenotypic traits+SSR	UPGMA+LDSS	25%	NT-SY Spc	(Li et al., 2012)
Forest trees	*Robinia pseudoacacia* L.	phenotypic traits+SSR	Multi-objective optimization across multiple software systems	32%	SSR:PowerMarker Core Hunter Phenotypic:PowerCore	(Guo et al., 2022)

### 4.3 Economic benefits and success cases of translating core germplasm into breeding applications

Core collections have demonstrably enhanced germplasm management and utilization, with their successes closely aligned with the methodological evolution of the field. This progression has systematically lowered the barriers between genetic resource conservation and practical breeding outcomes. The value of core germplasm is most concretely realized in its direct contribution to cultivar improvement. Phenotype-based cores provided filtered pools for traditional cultivar selection. Marker-defined mini-core collections enabled the identification and utilization of valuable alleles underlying key traits, such as drought tolerance in chickpea (Kashiwagi et al., 2005, 2013; Schafleitner et al., 2021), high oleic acid in peanut (Chu et al., 2007), and disease resistance in pepper (Brindisi et al., 2023). Currently, genomics-enabled core collections function as powerful association mapping panels for trait dissection (e.g., powdery mildew resistance in cucumber) and form the genetic basis for large-scale resequencing projects that define species-level diversity (e.g., the 3000 Rice Genomes Project, Rellosa et al., 2014; Wei et al., 2021).

Several landmark cases exemplify this translation from curated diversity to agricultural impact. In potato, the International Potato Center (CIP), in collaboration with Chinese researchers, utilized wild resources from the Andean center of origin to build a core evaluation population, leading to the development of the late blight-resistant variety “Cooperation 88” (C88). Widely adopted in southern China, this variety generated economic benefits exceeding $350 million by 2010, significantly reducing production risks for smallholder farmers (Li et al., 2011; Website CGIAR Genebank Initiative, 2019). For Cassava, Kasetsart University in Thailand collaborated with CIAT. Through screening a core collection, they introgressed the genetic background of a Venezuelan landrace (CMC 76) to develop “Kasetsart 50” (KU 50). This variety not only occupies over 56% of the planting area in Thailand but has also been adopted in Vietnam and other Southeast Asian countries, with cumulative economic contributions exceeding $97 million (Website CGIAR Genebank Initiative, 2019). In rice, the establishment of a “core germplasm–genomic analysis–trait association” paradigm facilitated the breeding of high-yielding varieties like ‘Huanghuazhan’, cultivated on millions of hectares, through the precise analysis of selection signatures and core haplotypes (Chen et al., 2017).

### 4.4 Systemic bottlenecks in germplasm resource management: redundancy, human error, and taxonomic bias

Despite significant achievements, global germplasm resource management faces severe challenges. First, the exponential growth of germplasm collections has led to a redundancy and inefficiency problem: the FAO’s Third Report on the State of the World’s Plant Genetic Resources for Food and Agriculture (2025) notes that while 1,750 genebanks worldwide conserve 7.4 million accessions, only about 25%-30% are genetically unique. A redundancy rate of approximately 70% imposes substantial financial and maintenance burdens (Anglin et al., 2025). Furthermore, “human error” poses a potential threat to the integrity of core collections. Research by Chen et al. (2016) indicated that during long-term seed regeneration cycles, about 20% of resources might suffer from taxonomic misidentification or genetic mixing due to labeling errors, pollen contamination, or operational mistakes. SNP validation techniques reveal that even in well-managed genebanks, species misidentification rates can reach 3.1%. Third, taxonomic preference in research focus also limits the field’s development. Current bibliometric results show that core germplasm research is heavily concentrated on field crops (rice, wheat, maize) and some legumes, while forest trees, medicinal plants, and “orphan crops” of high ecological and economic value remain marginalized (Ambati et al., 2020). Constructing core collections for medicinal plants is particularly complex, requiring consideration of both genetic diversity and “chemotypic” diversity, for which a robust theoretical framework is still lacking (Anjani et al., 2018; Wang et al., 2025).

### 4.5 Germplasm flows in the context of geopolitical dynamics: the crisis of multilateralism post-lima conference

The systemic bottlenecks identified in Section 4.4—data fragmentation and taxonomic bias—are not merely technical failures but are deeply intertwined with the current fragmentation of global governance and geopolitical dynamics.

The translational potential of core germplasm is fundamentally constrained by significant systemic and institutional barriers that exist beyond the laboratory. A core paradox lies in the disconnect between visible scientific collaboration and underlying data inaccessibility. While international co-authorship networks are well-established, the high-dimensional phenotypic and genotypic data generated through these studies often remain non-FAIR (Findable, Accessible, Interoperable, Reusable). This data fragmentation creates persistent silos, drastically reducing the global utility and cumulative impact of these valuable resources.

This technical challenge is compounded by a deeper, unresolved governance crisis at the international policy level. The scientific utility of core germplasm is directly threatened by the current geopolitical stalemate over genetic resource access and benefit-sharing. The failure to reach consensus on modernizing the Multilateral System (MLS) of the International Treaty on Plant Genetic Resources for Food and Agriculture during its Eleventh Governing Body session in Lima (GB-11, 2025) highlights a critical impasse (Website Pro-Wild., 2025). The core dispute centers on the governance of “Digital Sequence Information” (DSI). Technologically advanced nations advocate for maintaining open access to DSI as essential for innovation, while biodiversity-rich countries argue that DSI represents a “dematerialized” form of genetic resources and demand mandatory benefit-sharing mechanisms (e.g., the “Cali Fund”) to prevent “digital biopiracy.” (Website Dinesh Abrol, 2025). This unresolved legal vacuum not only perpetuates data siloing by eroding the trust necessary for open sharing but also actively hinders the transnational exchange of digital germplasm resources urgently needed to develop climate-resilient crops (Website Marcel Bruins, 2025).

## 5 Research prospects

In the future, plant core germplasm research must transcend mere “descriptive sampling” and achieve deep integration with “functional design” and “intelligent prediction.”

### 5.1 Building AI-driven biodigital resource centers

Future genebanks will evolve from simple “seed warehouses” into “Biodigital Resource Centers (BRCs)” (Yuan et al., 2025). In this new paradigm, each core accession will possess its “digital twin”—a holistic dataset comprising deep-coverage whole-genome sequences, multi-omics data (transcriptomics, metabolomics), and dynamic imaging data (Yuan et al., 2025). Artificial Intelligence will act as a “digital curator” in resource management. Leveraging Large Language Models (LLMs) and multimodal AI, systems will autonomously detect taxonomic anomalies, predict seed viability, and recommend parental combinations with high breeding potential based on literature mining (Stenhouse et al., 2025). In core collection selection algorithms, machine learning will replace traditional heuristic searches, utilizing algorithms like NSGA-II to find optimal trade-offs within feature spaces encompassing tens of thousands of dimensions (Gouy et al., 2025; Yuan et al., 2026).

### 5.2 Full integration of high-throughput phenomics and computer vision technologies

The “phenotyping bottleneck” has long constrained the precise evaluation of core collections. In the future, deep learning-based computer vision technologies (e.g., YOLO11, CNN) will enable automated, non-destructive trait extraction (Zhao et al., 2025). Through drone swarms, mobile phenotyping platforms, and smartphone video streams, researchers can capture the dynamic responses of core populations throughout the entire growing season in real-time (Araus et al., 2018). This dynamic monitoring capability is particularly crucial for climate change adaptation. For example, research on the Northern European Barley Core Collection (ExHIBiT) demonstrates that longitudinal GWAS analysis can identify key alleles associated with water-use efficiency and flood resilience—insights unattainable through static, one-time evaluations (Bernad et al., 2024).

### 5.3 Constructing functional and chemical core collections through interdisciplinary lenses

The design objective for core collections should shift from “general representativeness” to “goal-oriented” frameworks. Chemical Core Collections (CCCs): For medicinal and health-food plants (MHFPs), future research should prioritize the “metabolome” as the core sampling metric (Wang et al., 2025). Techniques like UPLC-Q-TOF-MS/MS will be used to dissect the variation patterns of secondary metabolites, constructing core sets that maximally cover specific active compounds (e.g., alkaloids, flavonoids, volatile oils). This ensures the authenticity and quality stability of medicinal materials (Chen et al., 2025a). Landscape Genomics-Guided Core Sets: For forest trees and crop wild relatives, core collections should be constructed by integrating environmental variables (e.g., mean temperature, precipitation, altitude) to form “landscape core collections” (Zhang et al., 2023). By estimating the “genomic offset,” populations with the highest adaptive potential under future climate scenarios can be selected, providing precise seed sources for ecological restoration (Labiszak and Wachowiak, 2025).

### 5.4 The leap from descriptive repositories to precision predictive breeding platforms

The ultimate vision of core germplasm research is to achieve “predictive breeding.” Utilizing pan-genome graphs, future core collections will no longer be constrained by a single reference genome but will encompass the species’ entire “pan-allelic library.” (Krusenbaum and Wissuwa, 2025). Integrating whole-genome prediction (GWP) with gene-editing technologies like CRISPR, researchers can simulate the performance of different genotypes under various environmental combinations directly within the digital models of core collections. This enables “*de novo* domestication” of selected target loci (Razzaq et al., 2021). This shift from “discovery” to “creation” will transform core germplasm into the ultimate engine for driving sustainable agriculture and global food security.

## Data Availability

The original contributions presented in the study are included in the article/**Supplementary Material**. Further inquiries can be directed to the corresponding authors.

## Supplementary material

Supplementary Table 1 Top 5 most cited Chinese publications on core collections.

Supplementary Table 2 Top 5 most cited English publications on core collections.

Supplementary Figure 1 Author collaboration network diagrams. (A) CNKI; (B) Web of Science (WoS).

Supplementary Figure 2 Institutional collaboration network diagrams. (A) CNKI; (B) Web of Science (WoS).
