# Supplementary material for: Scientific evolution and translational horizons of plant core germplasm: a global bibliometric synthesis and strategic insights
Source: Front Plant Sci. 2026 Mar 19;17:1771164. doi: 10.3389/fpls.2026.1771164 (PMC13044095; doi:10.3389/fpls.2026.1771164)
Supplement: Supplementary Table 1 — Top 5 most cited Chinese publications on core collections. [file Table1.docx]

| Author | Title | Journal | Citation Count | Year |
| --- | --- | --- | --- | --- |
| Hu et al. | Analysis on genetic diversity of phenotypic traits in rice (*Oryza sativa*) core collection and its comprehensive assessment | 《Acta Agronomica Sinica》 | 230 | 2012 |
| Wang et al. | Phenotypic diversity evaluations of *Foxtail millet* core collections | 《Acta Agronomica Sinica》 | 163 | 2016 |
| Chen et al . | Tea germplasm research in China： recent progresses and prospects | 《Journal of Plant Genetic Resources》 | 161 | 2004 |
| Qiu et al. | Establishment, representative testing and research progress of soybean core collection and mini core collection | 《Acta Agronomica Sinica》 | 151 | 2004 |
| Gao et al. | Development and evaluation of core collection of Japanese apricot germplasms in China | 《Scientia Agricultura Sinica》 | 126 | 2005 |

Supplementary Table 1 Top 5 most cited Chinese publications on core collections

# References

Chen, L.,Yang, Y. J.and Yu, F. L., (2004). Tea germplasm research in China:recent progresses and prospects. *J. Plant Genet. Res.* 8, 389–392. doi:10.13430/j.cnki.jpgr.2004.04.025

Hu, B. L.,Wan, Y., Li, X., Lei, J .G., Luo, X. D., Yan, W. G. et al. (2012). Analysis on genetic diversity of phenotypic traits in rice (*Oryza sativa*) core collection and its comprehensive assessment. *Acta Agron Sin.* 38, 829–839. doi:10.3724/SP.J.1006.2012.00829

Gao, Z. H., Zhang, Z., Han,Z.H., and Fang, J. G. (2005). Development and evaluation of core collection of Japanese apricot germplasms in China. *Scientia Agricultura Sinica.* 38, 363-368. <https://www.chinaagrisci.com/CN/10.3864/j.issn.0578-1752.at-2004-2082>

Qiu, L. J., Li, Y. H., Guan, R. X., Liu, Z. X., Wang, L. X., and Chang, R. Z., (2009). Establishment, representative testing and research progress of soybean core collection and mini core collection. *Acta Agron Sin.* 35, 571-579. doi:10.3724/SP.J.1006.2009.00571

Wang, H. G., Jia, G. Q., Zhi, H., Wen, Q. F., Dong, J. L., Chen, L., et al. (2016). Phenotypic diversity evaluations of *Foxtail Millet* core collections. *Acta Agron Sin.* 42, 19-30. doi:10.3724/SP.J.1006.2016.00019
