# Supplementary material for: Scientific evolution and translational horizons of plant core germplasm: a global bibliometric synthesis and strategic insights
Source: Front Plant Sci. 2026 Mar 19;17:1771164. doi: 10.3389/fpls.2026.1771164 (PMC13044095; doi:10.3389/fpls.2026.1771164)
Supplement: Supplementary Table 2 — Top 5 most cited English publications on core collections. [file Table2.docx]

Supplementary Table 2 Top 5 most cited English publications on core collections

| Author | Title | Journal | Citation Count | Year |
| --- | --- | --- | --- | --- |
| Uraguchi et al. | Root-to-shoot Cd translocation via the xylem is the major process determining shoot and grain cadmium accumulation in rice | 《Journal of Experimental Botany》 | 524 | 2009 |
| Rellosa et al. | The 3,000 rice genomes project | 《GigaScience》 | 326 | 2014 |
| Ma et al. | Resequencing a core collection of upland cotton identifies genomic variation and loci influencing fiber quality and yield | 《Nature Genetics》 | 311 | 2018 |
| Emanuelli et al. | Genetic diversity and population structure assessed by SSR and SNP markers in a large germplasm collection of grape | 《BMC Plant Biology》 | 270 | 2013 |
| Takahashi et al. | Variations in Hd1 proteins, Hd3a promoters, and Ehd1 expression levels contribute to diversity of flowering time in cultivated rice | 《Proceedings of the National Academy of Sciences of the United States of America》 | 233 | 2009 |

# References

Emanuelli, F., Lorenzi, S., Grzeskowiak, L., Catalano, V., Stefanini, M., Troggio, M., et al. (2013). Genetic diversity and population structure assessed bySSR and SNP markers in a large germplasm collection of grape. *BMC Plant Biol.* 13, 39. doi:10.1186/1471-2229-13-39

Ma, Z.Y., He, S.P., Wang, X.F., Sun, J.L., Zhang, Y., Zhang, G.Y., et al. (2018). Resequencing a core collection of upland cotton identifies genomic variation and loci influencing fiber quality and yield. *Nat. Genet.* 50, 803. doi:10.1038/s41588-018-0119-7

Rellosa, M.C., Reaño, R.A., Capilit, G., de Guzman, F.C., Ali, J., Hamilton, N., et al. (2014). The 3,000 rice genomes project. *Gigascience.* 3, 7. doi:10.1186/2047-217X-3-7

Takahashi, Y., Teshima, K. M., Yokoi, S., Innan, H., and Shimamoto, K. (2009). Variations in Hd1 proteins, Hd3a promoters, and Ehd1 expression levels contribute to diversity of flowering time in cultivated rice. *Proc. Natl. Acad. Sci. U. S. A.* 106, 4555-4560. doi:10.1073/pnas.0812092106

Uraguchi, S., Mori, S., Kuramata, M., Kawasaki, A., Arao, T., Ishikawa, S. (2009). Root-to-shoot cd translocation via the xylem is the major process determining shoot and grain cadmium accumulation in rice. *J. Exp. Bot.* 60, 2677-2688. doi:10.1093/jxb/erp119
