# Supplementary figures and images for: Scientific evolution and translational horizons of plant core germplasm: a global bibliometric synthesis and strategic insights
Source: Front Plant Sci. 2026 Mar 19;17:1771164. doi: 10.3389/fpls.2026.1771164 (PMC13044095; doi:10.3389/fpls.2026.1771164)

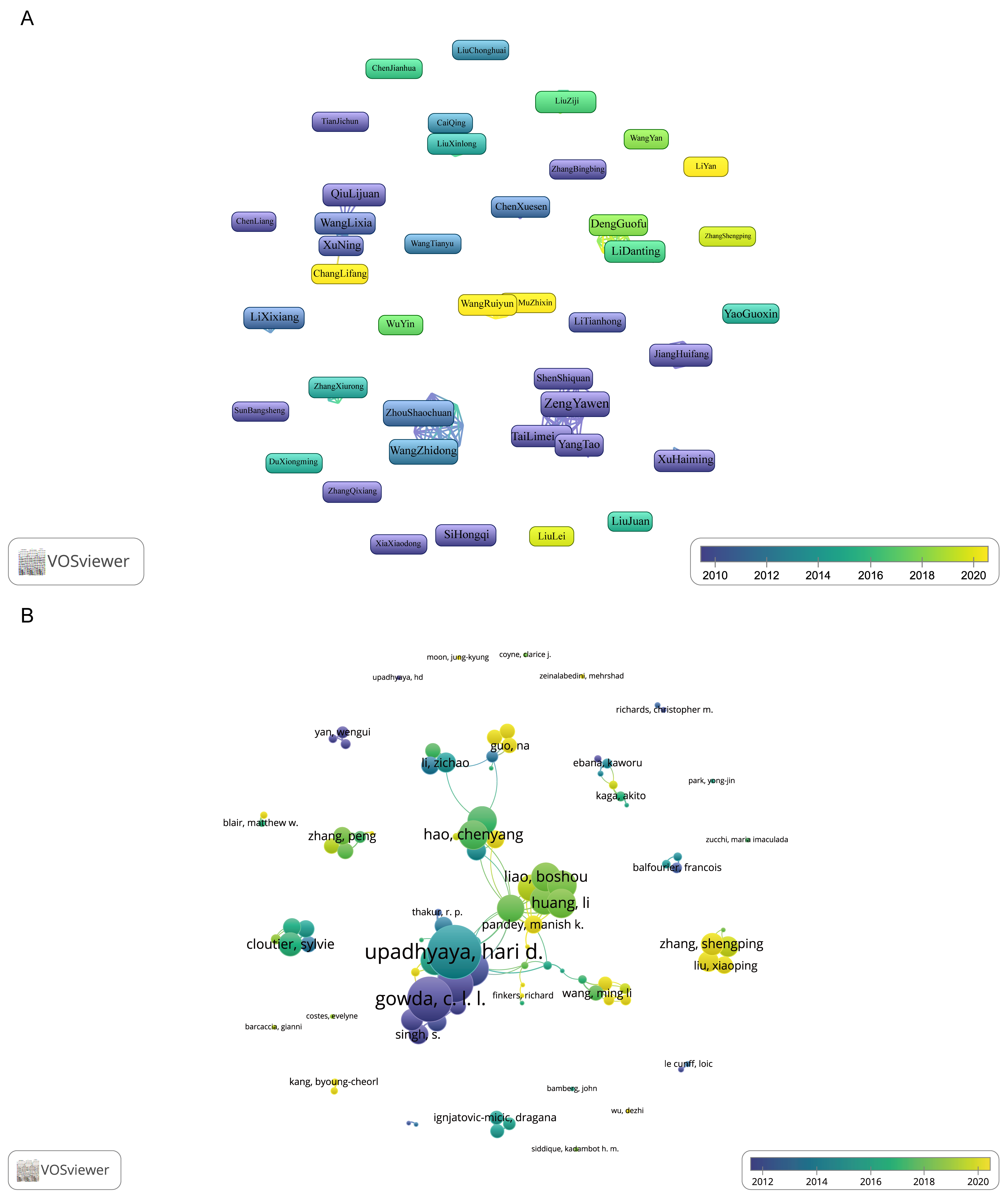

Supplement: Supplementary Figure 1 — Author collaboration network diagrams. (A) CNKI; (B) Web of Science (WoS). [file Image1.tif]

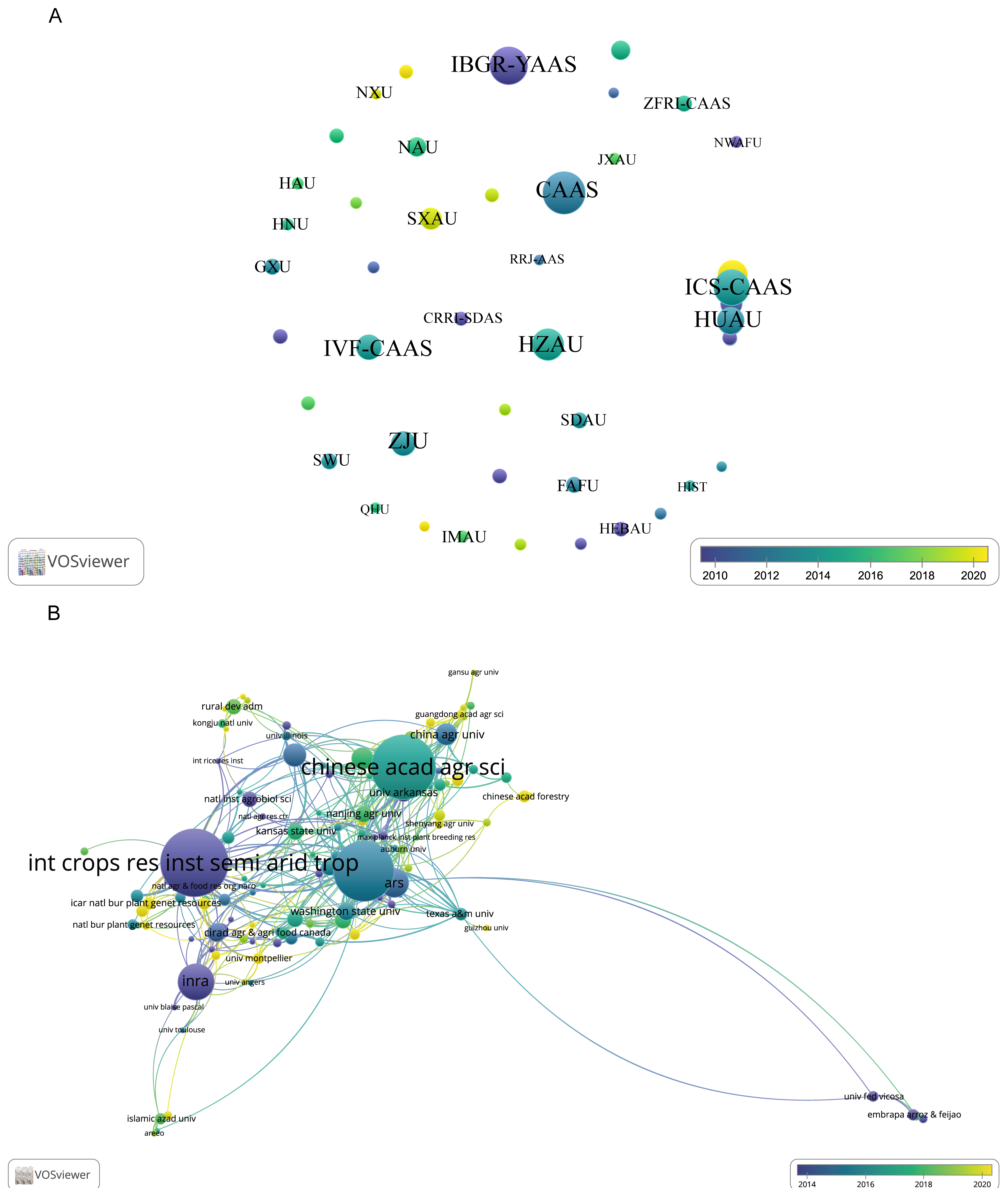

Supplement: Supplementary Figure 2 — Institutional collaboration network diagrams. (A) CNKI; (B) Web of Science (WoS). [file Image2.tif]
